# The genome and transcriptome of *Sarocladium terricola* provide insight into ergosterol biosynthesis

**DOI:** 10.3389/fcimb.2023.1181287

**Published:** 2023-04-14

**Authors:** Wei Wang, Yong Nie, Xiao-Yong Liu, Bo Huang

**Affiliations:** ^1^ Anhui Provincial Key Laboratory for Microbial Pest Control, Anhui Agricultural University, Hefei, China; ^2^ College of Life Sciences, Shandong Normal University, Jinan, China

**Keywords:** *Sarocladium terricola*, Phylogenomic analyses, genome, metabolites, transcriptome

## Abstract

*Sarocladium terricola* is a species of ascomycete fungus that has been recognized as a biocontrol agent for managing animal and plant pathogens, and exhibits significant potential as a feed additive. In this study, we utilized a combination of short-read Illumina sequencing and long-read PacBio sequencing to sequence, assemble, and analyze the genome of *S. terricola*. The resulting genome consisted of 11 scaffolds encompassing 30.27 Mb, with a GC content of 54.07%, and 10,326 predicted protein coding gene models. We utilized 268 single-copy ortholog genes to reconstruct the phylogenomic relationships among 26 ascomycetes, and found that *S. terricola* was closely related to two *Acremonium* species. We also determined that the ergosterol content of *S. terricola* was synthesized to nearly double levels when cultured in potato dextrose media compared to bean media (4509 mg/kg vs. 2382 mg/kg). Furthermore, transcriptome analyses of differentially expressed genes suggested that the ergosterol synthesis genes ERG3, ERG5, and ERG25 were significantly up-regulated in potato dextrose media. These results will help us to recognize metabolic pathway of ergosterol biosynthesis of *S. terricloa* comprehensivelly.

## Introduction

The genus *Sarocladium* W. Gams & D. Hawksw. (Sarocladiaceae, Hypocreales, Sordariomycetes, Ascomycota) is a diverse group of fungi proposed by Gams and Hawksworth in 1975, which includes important animal and plant pathogenic species (Gams and Hawksworth, 1975; Giraldo et al., 2015). *Sarocladium* comprises 30 species (http://www.indexfungorum.org, accessed on 13 November 13, 2022) with *S. oryzae* (Sawada) W. Gams & D. Hawksw as its type species (Gams and Hawksworth, 1975). Notably, *S. terricola* (J.H. Mill., Giddens & A.A. Foster) A. Giraldo, Gené & Guarro, originally classified as *Acremonium*, was transferred based on molecular evidence and morphological features (Giraldo et al., 2015).

*Sarocladium terricola* culture (well-known as *Acremonium terricola* culture or ATC) is an important feed additive, possessing various biological properties such as antioxidant, immunomodulatory, and anti-inflammatory activities (Li et al., 2016; Li et al., 2018). Previous studies suggested that ATC plays anti-inflammatory and antioxidant roles in lipopolysaccharide-induced mastitis in rats (Li et al., 2020), and improved rumen fermentation, decreased somatic cell counts in milk, and enhanced milk yield in cows or sheep when used as an animal feed additive (Jiang et al., 2018; Li et al., 2018; Kong et al., 2022). *S. terricola* is known to synthesize various metabolites, including D-mannitol, galactomannan, ergosterol, cordycepin, and essential amino acids (Li et al., 2020). Among these, ergosterol, an essential component of fungal cell membranes that plays an important role in maintaining structure and function, is the main focus of this study (White et al., 1998; Abe et al., 2009; Guan et al., 2009). Ergosterol is a clinically available target for most antifungal agents, and it exhibits anti-inflammatory and antioxidant properties (Dopont et al., 2012; Dhingra and Cramer, 2017). Additionally, the content of ergosterol in *S. terricola* is higher than other metabolites, making it a potential main contributor to the anti-inflammatory activity of ATC.

As the cost of sequencing has decreased, the number of species with genome sequence assemblies has rapidly increased. For example, the NCBI database contains more than 2,770 genomes from Ascomycota (https://www.ncbi.nlm.nih.gov/genome/, accessed on November 13, 2022). Genome and transcriptome sequences have provided novel insights into fungal phylogenomics, biosynthesis of metabolites, mechanisms of symbiosis, and others (Zhao et al., 2010; Ren et al., 2021; Sun et al., 2022; Wu et al., 2022; Zhao et al., 2022b; Zhao et al., 2022a). Although genome and mitochondrial genome sequences in the genus Sarocladium have been published (Hittalmani et al., 2016; Yao et al., 2016; Tian et al., 2022), phylogenomic relationships, transcriptome sequences, and characterization of the ergosterol biosynthesis pathway have not been studied. Transcriptome analyses mainly study gene transcription and regulation, revealing the molecular mechanisms of specific biological processes, such as symbiotic interactions between fungi and plants (Ren et al., 2021).

In this study, we generated a high-quality genome sequence assembly of *S. terricola* and characterized the *S. terricola* ergosterol biosynthesis pathway using transcriptome analyses combined with high performance liquid chromatography (HPLC).

## Materials and methods

### Strains, media, and fermentation

In this study, *Sarocladium terricola* was isolated from soil in Anhui Province, China, and deposited in the Anhui Agricultural University with the accession number RCEF 6201. The morphological trait is mainly as followed: Mycelia hyaline, 1.5–1.9 µm wide; Phialides subulate, solitary, 12–24 µm long, 2–3 µm wide at the base; Conidia fusiform, hyaline, sharply, 7–9 × 3–4 µm; Chlamydospores not observed.

Strains were incubated with potato dextrose agar media (PDA: potato 200 g/L, glucose 20 g/L, agar 20 g/L, and 1 L distilled water) at 25°C for 1 week to produce spores and then collected using sterile water. The spore suspension was adjusted to a concentration of 1 × 10^6^/mL, then 1 mL spore suspension was inoculated into a 500 mL flask containing either 200 mL potato dextrose media (potato 200 g/L, glucose 20 g/L, and 1 L distilled water) or bean media (bean 30 g/L, glucose 20 g/L, and 1 L distilled water). Finally, the two types of media including HCBe (cluturing in bean media) and HCPo (cluturing in potato dextrose media) with difference in nitrogen source were incubated at 25°C with 150 rpm shaking for three days, and ergosterol was then detected using high performance liquid chromatography (HPLC). Three biological replicates were performed for each type of media.

### Ergosterol measurement

We referred and improved the previous study (Nout et al., 1987): after three days, fresh mycelium was collected using a circulating water multi-purpose vacuum pump and placed in an oven at 45°C and allowed to equilibrate. To extract ergosterols, 0.5 g mycelium was mixed with 50 mL methanol in a 100 mL flask, disrupted with ultrasonic waves at 60°C for 30 min, and then centrifuged at 4000 g for 5 min to obtain supernatant. As a standard, 0.01 g ergosterol was placed in a 100 mL volumetric flask with methanol solvent added until volume reached 100 mL.

The ergosterol profiles were assayed using HPLC with a C18 column (250 mm × 4.6 mm × 5 µm). The analytical conditions were as follows: solvent system, anhydrous methanol; temperature, 35°C; injection volume, 10 µL; flow rate, 1 mL/min; and UV wavelength, 268 nm. Three technical replicates were assayed. The amount of ergosterol in the sample was determined by mass fraction (ω, unit is expressed in mg/kg), and the formula used for calculation was as follows:


$$\omega =\frac{\text{A }\times \text{ ρS }\times \text{ V}}{\text{As }\times \text{ m }}\times \text{f}$$

A: peak area of ergosterol in sample.

As: peak area of ergosterol in standard working solution.

ρS  : mass concentration of ergosterol in standard working solution (unit: μg/mL).

V: final constant volume of sample solution (unit: mL).

m: mass of the test portion (unit: g).

f: sample dilution ratio.

### Genome sequencing and assembly, gene prediction, and functional annotation

*S. terricola* was incubated in potato dextrose agar media (PDA: potato 200 g/L, glucose 20 g/L, agar 20 g/L, and 1 L distilled water) and cultured for two weeks. Total cell DNA was extracted from mycelia using methods as described in a previous study (Zhao et al., 2022b) and detected using the DNA/Protein Analyzer and 1% agarose gel electrophoresis. Two approaches were used for sequencing: paired-end short reads (300 bp) using the Illumina NovaSeq 6000 platform and long reads (more than 20 kb) using the PacBio Sequel II platform. Low-quality reads were removed (end-polished, A-tailed, and ligated with the full-length adaptor for Illumina sequencing, and short reads less than 1000 bp for PacBio sequencing), leaving only high-quality reads. The high-quality reads were *de novo* assembled using SMRT (version 5.1.0) downloads from https://www.pacb.com/support/software-downloads with default parameters (Chin et al., 2013) and assessed using BUSCO (version 5.2.2) with Ascomycota gene set downloaded from https://busco-data.ezlab.org/v4/data/lineages/ascomycota_odb10.2020-09-10.tar.gz (Simão et al., 2015).

Gene prediction was performed using four tools, Augustus (version 3.2.1) (Stanke et al., 2008), Genemark-ES (version 4.21) (Ter-Hovhannisyan et al., 2008), Genewise (version 2.20) (Birney et al., 2004), and SNAP (version 2010-07-28) (Johnson et al., 2008), and EvidenceModeler (version 1.1.1) (Haas et al., 2008) was then used to make integrated gene models. A total of ten databases were used to functionally annotate genes, namely Gene Ontology (GO) (Ashburner et al., 2000), Kyoto Encyclopedia of Genes and Genomes (KEGG) (Kanehisa et al., 2016), Cluster of Orthologous Groups of proteins (COG) (Galperin et al., 2015), Swiss-Prot (Consortium, 2015), nr (https://www.ncbi.nlm.nih.gov/protein/), Pathogen Host Interactions (PHI) (Torto-Alalibo et al., 2009), Fungal Cytochrome P450 (Fischer et al., 2007), Carbohydrate-Active Enzymes (CAZy) (Levasseur et al., 2013), Virulence Factor Database (VFDB) (Chen et al., 2016), and Type III secretion system Effector protein (T3SS) (Vargas et al., 2012) using Diamond with an e-value less than 1 × 10^−5^. In addition, antiSMASH (fungal version) (Medema et al., 2011), Extensive *de novo* TE Annotator (EDTA) pipeline (version 1.9.5) (Ou et al., 2019), RNAmmer (version 1.2) (Lagesen et al., 2007), and tRNAscan-SE (version 2.0.5) (Lowe and Eddy, 1997) were used to predict gene clusters of secondary metabolites, repetitive elements, rRNAs, and tRNAs, respectively.

### Phylogenomic analysis

In this study, a total of 26 species of ascomycetes (**Table S1**), including 25 genomes downloaded from genomic databases on the NCBI website and the strain of *S. terricola* (RCEF 6201) sequenced in this study, were used for phylogenomic analysis. This was performed using single copy ortholog genes that were identified using OrthoFinder (version 2.5.4) (Emms and Kelly, 2019) and aligned using MAFFT (version 7) with default parameters (Katoh and Standley, 2013). A Maximum Likelihood (ML) tree was reconstructed with RaxML (version 8.2.12) (Stamatakis, 2014) using the best optimal model of PROTGAMMAILGF with 100 bootstrap replicates.

### Transcriptome sequencing and assembly

Six samples (three biological replicates from both types of growth media) of *S. terricola* were collected, and total RNA was extracted and purified using methods as described in a previous study (Ren et al., 2021). The transcriptome libraries were constructed and sequenced using the Illumina NovaSeq 6000 platform. After quality control and cleaning of raw reads, the high-quality reads were aligned and assembled using HISAT2 (version 0.1.6-beta) (Kim et al., 2015) and Cufflinks (version 2.2.1) (Trapnell et al., 2012), respectively.

### Differential gene expression and enrichment analyses

Bowtie2 (version 2.2.5) (Langmead and Salzberg, 2012) was used to count the proportion of high-quality reads of each sample gene compared to the genome of *S. terricola*, and RSEM (version 1.2.12) (Li and Dewey, 2011) was then used to convert to standardized gene expression levels, viz. reads per kilobase of exon model per million mapped reads (FPKM values). DESeq2 (Wang et al., 2009) was used to identify differentially expressed genes (DEGs), using the criteria of fold change (FC) ≥ 2 and false discovery rate (FDR)< 0.05. Functional enrichments of DEGs were performed using Gene Ontology (GO) and Kyoto Encyclopedia of Genes and Genomes (KEGG) pathway databases.

## Results

### Ergosterol content in different media

**Figure 1:**
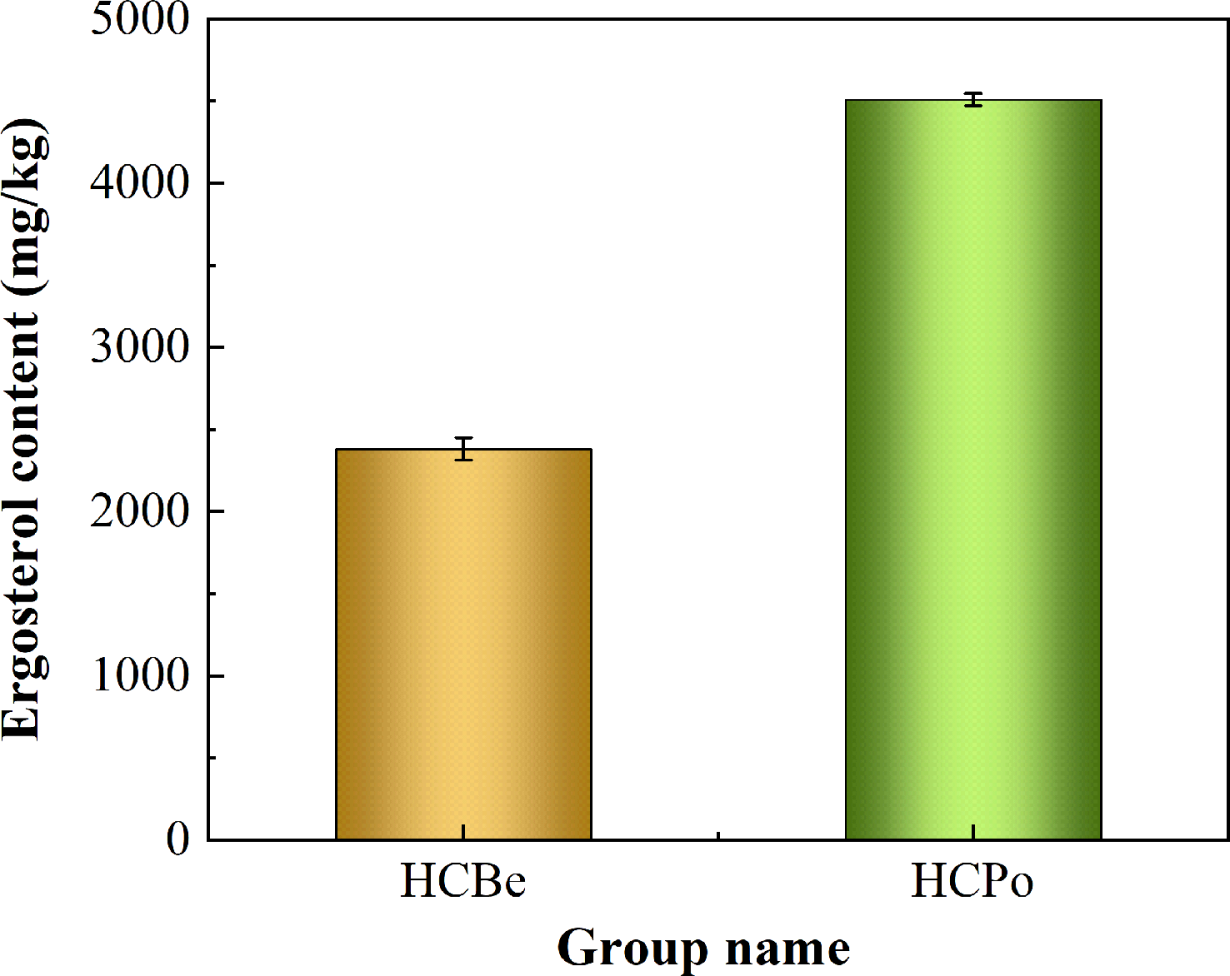
The ergosterol content in two types of media. Notes: HCBe, bean media; HCPo, potato dextrose media.

The present study measured the ergosterol content in *Sarocladium terricola* grown in two different growth media: potato dextrose media and bean media. The study found that ergosterol synthesis was more efficient in potato dextrose media, which resulted in a significantly higher content of ergosterol compared to bean media. Specifically, the ergosterol content in potato dextrose media was 4509 mg/kg, while in bean media, it was only 2382 mg/kg. This difference in ergosterol content between the two growth media was statistically significant with a p-value less than 0.01, as shown in **Figure 1**.

### Genomic characteristics of the *S. terricola* assembly

**Figure 2:**
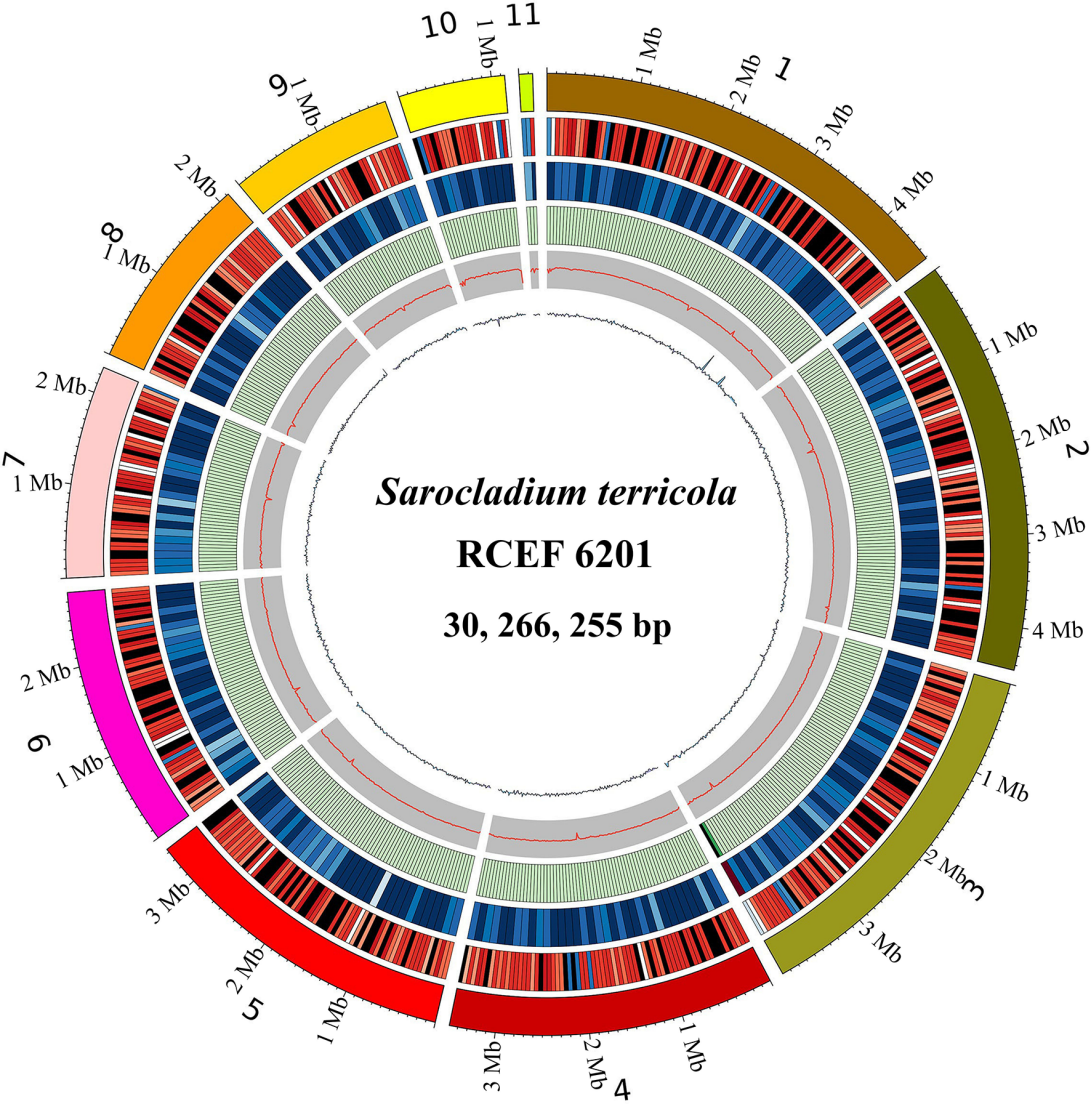
Genome features of *Sarocladium terricola* (RCEF6201). From outer to inner: 1: Genome scaffolds (sorted by length); 2: Gene density (in 50000bp nonoverlapping windows); 3: ncRNA density (in 100000bp nonoverlapping windows); 4: Repeats coverage (in 50000bp nonoverlapping windows); 5: GC content (in 20000bp nonoverlapping windows); 6: GC skew (in 20000bp nonoverlapping windows).

The genome assembly of *S. terricola* spans 11 scaffolds and contains 30.27 Mb with a GC content of 54.07% (**Table 1**; **Figure 2**). We predicted a total of 10,326 protein-coding gene (PCG) models using four gene prediction tools. Among these gene models, 5,926, 4,220, 1,277, 3,044, 9,055, 1,425, 124, 321, 42, and 3,661 were functionally annotated using GO, KEGG, COG, Swiss-Prot, nr, PHI, P450, CAZy, VFDB, and T3SS databases, respectively. In addition, we predicted 41 gene clusters of secondary metabolites, including 9 NRPS-like, 11 NRPS, 12 T1PKS, 2 T2PKS, 5 terpene, and a single cluster of both beta-lactone and phosphonate. Repetitive elements accounted for 0.81% of the whole genome. Moreover, we detected 282 non-coding RNAs (ncRNAs) in this genome.

**Table 1:** Genomic features of *Sarocladium terricola* sequence assembly.

Species		*Sarocladium terricola* (RCEF6201)
Genome size (Mb)		30.27
Scaffolds		11
Largest scaffolds (Mb)		4.61
GC (%)		54.07
N50 (Mb)		3.49
L50		4
Assembly BUSCO coverage (%)		96.7
PCG models		10,326
	GO	5,926
	KEGG	4,220
	COG	1,277
	Swiss-Prot	3,004
	nr	9,055
	PHI	1,425
	P450	124
	CAZy	321
	VFDB	42
	T3SS	3,661
Gene clusters of secondary metabolites		
	NRPS-like	9
	NRPS	11
	T1PKS	12
	T3PKS	2
	Terpene	5
	Beta-lactone	1
	Phosphonate	1
	Total	41
Repetitive elements (% in genomes)		
	LTR	0.25%
	TIR	0.50%
	nonTIR	0.06%
	Total	0.81%
ncRNA		
	rRNA	39
	tRNA	103
	sRNA	45
	snRNA	20
	miRNA	75

### Phylogenomic analysis

**Figure 3:**
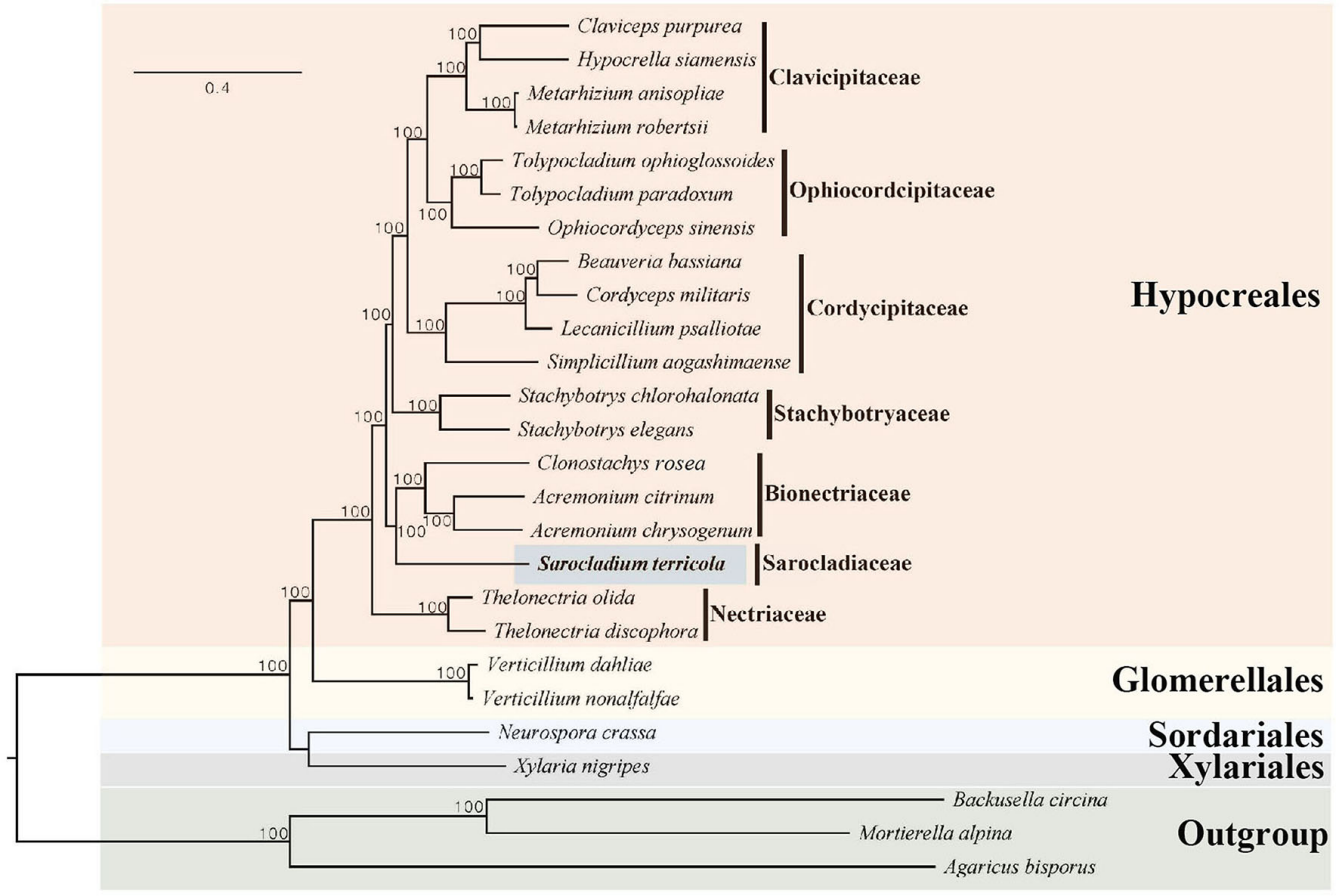
Maximum Likelihood phylogenomic tree based on the amino acids encoded by 268 single copy ortholog genes from 26 ascomycetes. The new genome sequence from the present study is highlighted in blue. Maximum Likelihood bootstrap values (≥ 95%) are indicated along branches. A scale bar in the upper left indicates substitutions per site.

A phylogenomic tree was reconstructed using single copy ortholog genes from 26 species, with *Agaricus bisporus*, *Mortierella alpina*, and *Backusella circina* serving as outgroups. A total of 268 single copy ortholog genes were identified through OrthoFinder (Emms and Kelly, 2019), and were used to create an alignment dataset with a length of 339,589 characters. In the Maximum Likelihood phylogenomic tree, *S. terricola* of the family Sarocladiaceae was found to be within a clade that included members of the family Bionectriaceae, such as *Acremonium chrysogenum* and *A. citrinum* (refer to **Figure 3**).

### Transcriptome sequence analyses and differentially expressed genes

To investigate the impact of ergosterol biosynthesis in different media, we performed transcriptome analyses of *S. terricola*. We sequenced six transcriptome libraries, three from both bean and potato dextrose media, resulting in a total of 37,043,298 to 42,033,580 clean reads per sample. A total of 5.56 Gb to 6.31 Gb of clean reads were generated, with more than 95.93% of clean reads meeting the Q30 quality threshold (**Table S2**). Furthermore, over 97.65% of the clean reads mapped to the genome of *S. terricola*. In each sample, a total of 9,176-9,738 genes (88.86-94.31% of the total number of genes) were found to be expressed.

**Figure 4:**
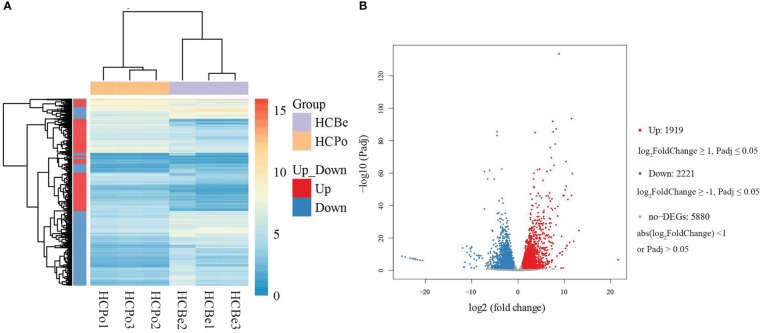
The differentially expressed genes of *S. terricola* from two types of media. **(A)** The heat map of expression levels of differentially expressed genes, **(B)** Volcano plot for HCBe vs. HCPo based on the DESeq2 method. “Up” and “Down” refer to levels in potato dextrose media as compared to bean media.

**Figure 5:**
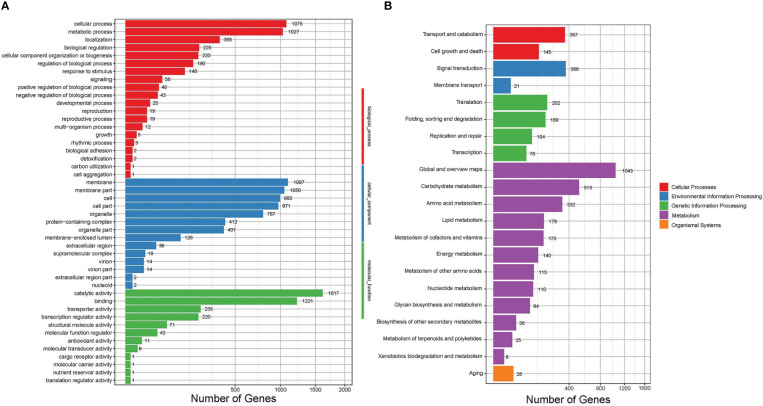
GO function and KEGG pathway enrichment analyses for DEGs of *Sarocladium terricola* cultured in two types of media. **(A)** GO analysis, **(B)** KEGG analysis. Differentially expressed genes were classified into biological process, cellular component, and molecular function categories in the GO analysis, and cellular processes, environmental information processing, genetic information processing, metabolism and organismal systems in the KEGG analysis.

The differential expression analysis indicated that the HCBe group had a slightly higher number of down-regulated genes compared to up-regulated genes (2221 vs. 1919) when compared to the HCPo group. Furthermore, 5880 genes did not exhibit any significant differences in their expression levels between the two groups, as depicted in **Figure 4**. To gain insights into the biological functions of the differentially expressed genes, we performed GO and KEGG pathway enrichment analyses, which are shown in **Figures 5**, **S1**. Nineteen GO categories were significantly enriched with over 100 DEGs, including cellular processes, metabolic processes, membrane, membrane parts, catalytic activity, and binding, as illustrated in **Figures 5A**, **S1A**. Additionally, the KEGG pathway enrichment analysis identified several major pathways associated with the set of DEGs, including global and overview maps, carbohydrate metabolism, signal transduction, cell growth and death, and amino acid metabolism, as shown in **Figures 5B**, **S1B**.

### Ergosterol biosynthesis in *S. terricola*

**Figure 6:**
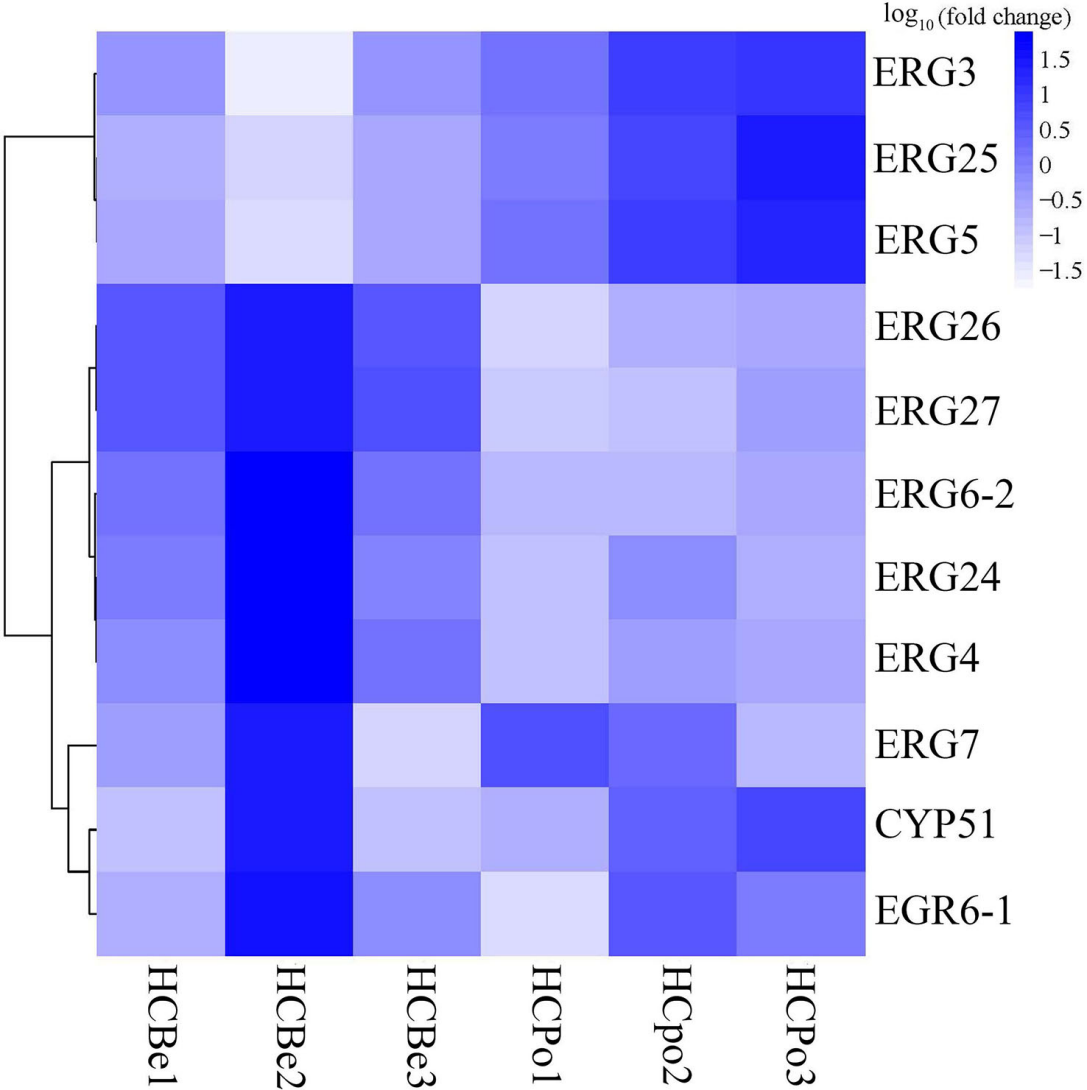
The heat map of expression levels of genes associated with ergosterol biosynthesis in *Sarocladium terricola*. ERG3 (S7G08143, delta7-sterol 5-desaturase), ERG25 (S5G06091, methylsterol monooxygenase), ERG5 (S5G06474, sterol 22-desaturase), ERG26 (S2G02191, sterol-4alpha-carboxylate 3-dehydrogenase), ERG27 (S7G08361, 3-keto steroid reductase), ERG6-2 (S1G00302, sterol 24-C-methyltransferase), ERG24 (S3G03213, delta14-sterol reductase), ERG4 (S1G00326, delta24 (24(1))-sterol reductase), ERG7 (S2G02520, lanosterol synthase), CYP51 (S1G01142, sterol 14alpha-demethylase), and ERG6-1 (S10G10199, delta24(24(1))-sterol reductase). Fold change refers to expression levels in potato dextrose media as compared to bean media.

In this study, we annotated eleven genes (**Figure 5**) associated with ergosterol biosynthesis from the KEGG database, which were lanosterol synthase (ERG7; S2G02520), sterol 14alpha-demethylase (CYP51; S1G01142), delta7-sterol 5-desaturase (ERG24; S3G03213), methylsterol monooxygenase (ERG25; S5G06091), sterol-4alpha-carboxylate 3-dehydrogenase (ERG26; S2G02191), 3-keto steroid reductase (ERG27; S7G08361), sterol 24-C-methyltransferase (ERG6-1: S10G10199 and ERG6-2: S1G00302), Delta7-sterol 5-desaturase (ERG3; S7G08143), sterol 22-desaturase (ERG5; S5G06474), and delta24(24(1))-sterol reductase (ERG4; S1G00326). Out of these genes, five genes (S3G03213, S2G02191, S1G00302, S1G00326, and S7G08361) were significantly down-regulated in the HCPo group, whereas three genes (S5G06474, S5G06091, and S7G08143) were significantly up-regulated, as shown in **Figure 6**.

## Discussion

With the advent of genome and transcriptome sequencing, omics technology has been increasingly utilized to investigate various biological processes, discover novel natural products, and reconstruct phylogenomic relationships (Hittalmani et al., 2016; Ren et al., 2021; Tian et al., 2022; Wu et al., 2022; Zhao et al., 2022b; Zhao et al., 2022a). Although the *Sarocladium* genus encompasses 30 species (http://www.indexfungorum.org, accessed on 13 November 2022), genome-scale studies of *Sarocladium* remain scarce. The first genome sequence analysis of *Sarocladium oryzae*, a pathogen causing sheath rot disease in rice, identified genes associated with helvolic acid and cerulenin biosynthesis pathways and identified approximately 9.37% of *S. oryzae* genes as pathogenicity genes (Hittalmani et al., 2016). In the present study, we identified 1,425 genes from the S. terricola genome assembly in the Pathogen Host Interactions (PHI) database, accounting for 13.8% of predicted protein-coding gene models (**Table 1**). This finding implies that *Sarocladium* species harbor a vast array of pathogenic genes. Moreover, *S. oryzae* and *S. terricola* have been reported to contain numerous natural biosynthetic gene clusters (Hittalmani et al., 2016; Tian et al., 2022). For example, 33 secondary metabolite gene clusters were discovered in the S. terricola strain TR, including ten NRPS clusters, thirteen PKS clusters, one PKS-NRPS hybrid cluster, and five terpene clusters (Tian et al., 2022). This study identified slightly more gene clusters in the *S. terricola* genome assembly (**Table 1**).

Ergosterol is a crucial component of fungal cell membranes and has numerous biological and physiological functions, including delaying aging, preventing cancer, reducing inflammation and fever, and exhibiting anti-oxidative and antibacterial activities (Fernandes and Cabral, 2007; Abe et al., 2009; Guan et al., 2009; Caspeta et al., 2014; Dhingra and Cramer, 2017). Although fungi can accumulate ergosterol, its content is influenced by several factors, such as biomass, growth temperature, oxygen concentration, and the availability of carbon and nitrogen sources (Shang et al., 2006; He et al., 2007). In this study, significant differences in ergosterol content were observed when *S. terricola* was cultured in different media, including bean and potato dextrose media. To investigate the mechanism of ergosterol biosynthesis, we analyzed gene expression using transcriptome sequences from cultures grown in different media. Our analysis revealed that ERG3, ERG5, and ERG25 were up-regulated in potato dextrose media compared to bean media, while ERG4, ERG6, ERG24, ERG26, and ERG27 were down-regulated. In *Saccharomyces cerevisiae*, the enzymes essential for ergosterol biosynthesis include ERG9, ERG1, ERG7, ERG11, ERG24, ERG25, ERG26, ERG27, ERG6, ERG2, ERG3, ERG4, and ERG5 (Hu et al., 2018). The catalytic steps of ERG3, ERG5, and ERG25 require oxygen (Onyewu et al., 2003). Therefore, the up-regulation of ERG3, ERG5, and ERG25 in *S. terricola* grown in potato media may promote the synthesis of ergosterol. We also found no significant difference in the expression of three genes (S2G02520, S1G01142, and S10G10199). The three genes with significantly increased expression in potato dextrose media were ERG3, ERG25, and ERG5, which participate in the final steps of ergosterol synthesis and require the participation of oxygen. The down-regulated genes, ERG26, ERG27, and ERG6, have similar functions to ERG25, catalyzing the synthesis of fecosterol from 4,4-dimethylzymosterol. ERG4 and ERG5 participate in the synthesis of ergosta-5,7,24(28)-stienol, which is a precursor to ergosterol. Thus, the down-regulated genes, ERG26, ERG27, ERG6, and ERG4, have a limited effect in potato media.

To reconstruct the evolutionary history of *S. terricola*, we performed a maximum likelihood (ML) analysis using 268 single-copy orthologous genes from 26 genomes. The resulting ML tree revealed that *S. terricola* did not form a clade with *Acremonium chrysogenum* and *A. citrinum*, which supported the transfer of *A. terricola* to the genus *Sarocladium* (Giraldo et al., 2015).

## Conclusion

In the present study, we sequenced, assembled, and annotated the genome of *S. terricola*. Our HPLC results indicated that the ergosterol levels of *S. terricola* were almost twice as high when cultured in potato dextrose media compared to bean media. Furthermore, our transcriptome analyses suggested that ERG3, ERG5, and ERG25 were significantly up-regulated in potato dextrose media, which may contribute to the increased ergosterol levels. Our phylogenomic analysis demonstrated that *S. terricola* is closely related to the family Bionectriaceae. The findings of this study hold great promise for the application of *S. terricola* as an animal feed additive. Additionally, we plan to conduct further studies to explore the relative genes involved in ergosterol biosynthesis, with the aim of further enhancing the ergosterol content of this strain in future work.

## Data availability statement

The genomic and transcriptomic sequences have been deposited in GenBank with accession numbers: JAHKSO000000000 and PRJNA949268, respectively. 

## Author contributions

WW; conceptualization, writing—original draft preparation, and writing—review, YN; formal analysis, X-YL; investigation, BH; funding acquisition, projection administration, and writing—review. All authors contributed to the article and approved the submitted version.

## Supplementary material

Supplementary Figure 1 GO function and KEEG pathway enrichment analyses in responding to the two type media of *S. terricola*. **(A)** differentially expressed genes number of the most enriched GO term, **(B)** differentially expressed genes number of the most enriched KEGG pathway.
