# Supplementary material for: The genome and transcriptome of Sarocladium terricola provide insight into ergosterol biosynthesis
Source: Front Cell Infect Microbiol. 2023 Apr 14;13:1181287. doi: 10.3389/fcimb.2023.1181287 (PMC10140317; doi:10.3389/fcimb.2023.1181287)
Supplement: Supplementary file 1 [file Table_1.docx]

Table S1. The genomic information used for phylogenomic analyses in this study.

| Species | Strains | BioSample | References |
| --- | --- | --- | --- |
| *Acremonium chrysogenum* | ATCC 11550 | SAMN02799700 | (Terfehr et al., 2014) |
| *Acremonium citrinum* | FKII-L8-BK-P5 | SAMN25226811 | - |
| *Agaricus bisporus* | - | SAMEA8562043 | - |
| *Backusella circina* | CGMCC 3.15908 | SAMN28403603 | (Zhao et al., 2022) doi: 10.1101/2022.07.05.498902 |
| *Beauveria bassiana* | ARSEF 2860 | SAMN02981247 | (Xiao et al., 2012) |
| *Claviceps purpurea* | 20.1 | SAMN2272775 | (Schardl et al., 2013) |
| *Clonostachys rosea* | 67-1 | SAMN03339152 | (Sun et al., 2015) |
| *Cordyceps militaris* | CM 01 | SAMN02981304 | (Zheng et al., 2011) doi: 10.1186/gb-2011-12-11-r116 |
| *Hypocrella siamensis* | MTCC 10142 | SAMN02711855 | (Agrawal et al., 2016) doi: 10.1186/s12864-016-2710-6 |
| *Lecanicillium psalliotae* | HWLR 35 | SAMN08038437 | - |
| *Metarhizium anispoliae* | ARSEF 549 | SAMN03268434 | - |
| *Metarhizium robertsii* | ARSEF 23 | SAMN02981260 | (Gao et al., 2011) doi: 10.1371/journal.pgen.1001264 |
| *Mortierella alpina* | CGMCC 20262 | SAMN29492047 | (Zhao et al., 2022) doi: 10.3390/jof8090891 |
| *Neurospora crassa* | OR 74A | SAMN02953583 | (Galagan et al., 2003) doi: 10.1038/nature01554 |
| *Ophiocordyceps sinensis* | ZJB 12195 | SAMN04550853 | (Li et al., 2016) doi: 10.1016/j.mimet.2016.06.025 |
| *Sarocladium terricola* | RCEF 6201 | SAMN19460271 | This study |
| *Simplicillium aogashimaense* | HWYR 21 | SAMN15062681 | - |
| *Stachybotrys chlorohalonata* | IBT 40285 | - | (Semeiks et al., 2014) doi: 10.1186/1471-2164-15-590. |
| *Stachybotrys elegans* | MPI-CAGE-CH-0235 | SAMN06296943 | - |
| *Tolypocladium ophioglossoides* | CBS 100239 | - | (Quandt et al., 2015) doi: 10.1186/s12864-015-1777-9 |
| *Tolypocladium paradoxum* | NRBC 100945 | SAMN08279508 | - |
| *Thelonectria discophora* | - | SAMEA8990552 | - |
| *Thelonectria olida* | MPI-CAGE-CH-0241 | SAMN06296308 | - |
| *Verticillium dahliae* | Getta Getta | SAMN10457093 | (Depotter et al., 2019) doi: 10.1111/mec.15168 |
| *Verticillium nonalfalfae* | WCS 072 | SAMN14679391 | (Kasson et al., 2019) doi: 10.1128/MRA.01619-18. |
| *Xylaria nigripes* | YMJ 653 | SAMN13172309 | (Franco et al., 2022) doi: 10.1111/nph.17873. |

[C Alisha Quandt](https://pubmed.ncbi.nlm.nih.gov/?term=Quandt+CA&cauthor_id=26215153) , [Kathryn E Bushley](https://pubmed.ncbi.nlm.nih.gov/?term=Bushley+KE&cauthor_id=26215153),[Joseph W Spatafora](https://pubmed.ncbi.nlm.nih.gov/?term=Spatafora+JW&cauthor_id=26215153).2015. The genome of the truffle-parasite Tolypocladium ophioglossoides and the evolution of antifungal peptaibiotics.BMC Genomics 16:553.

[Heng Zhao](https://pubmed.ncbi.nlm.nih.gov/?term=Zhao+H&cauthor_id=36135616), [Yong Nie](https://pubmed.ncbi.nlm.nih.gov/?term=Nie+Y&cauthor_id=36135616) , [Yang Jiang](https://pubmed.ncbi.nlm.nih.gov/?term=Jiang+Y&cauthor_id=36135616) , [Shi Wang](https://pubmed.ncbi.nlm.nih.gov/?term=Wang+S&cauthor_id=36135616) , [Tian-Yu Zhang](https://pubmed.ncbi.nlm.nih.gov/?term=Zhang+TY&cauthor_id=36135616) , [Xiao-Yong Liu](https://pubmed.ncbi.nlm.nih.gov/?term=Liu+XY&cauthor_id=36135616) .2022.Comparative Genomics of Mortierellaceae Provides Insights into Lipid Metabolism: Two Novel Types of Fatty Acid Synthase. J Fungi (Basel). 2022 Aug 23;8:891.

Heng Zhao, Yu-Cheng Dai, Xiao-Yong Liu.2022. Outline and divergence time of subkingdom Mucoromyceta: two new phyla, five new orders, six new families and seventy-three new species.

[James E Galagan](https://pubmed.ncbi.nlm.nih.gov/?term=Galagan+JE&cauthor_id=12712197) , [Sarah E Calvo](https://pubmed.ncbi.nlm.nih.gov/?term=Calvo+SE&cauthor_id=12712197), [Katherine A Borkovich](https://pubmed.ncbi.nlm.nih.gov/?term=Borkovich+KA&cauthor_id=12712197), [Eric U Selker](https://pubmed.ncbi.nlm.nih.gov/?term=Selker+EU&cauthor_id=12712197), [Nick D Read](https://pubmed.ncbi.nlm.nih.gov/?term=Read+ND&cauthor_id=12712197), [David Jaffe](https://pubmed.ncbi.nlm.nih.gov/?term=Jaffe+D&cauthor_id=12712197), [William FitzHugh](https://pubmed.ncbi.nlm.nih.gov/?term=FitzHugh+W&cauthor_id=12712197), [Li-Jun Ma](https://pubmed.ncbi.nlm.nih.gov/?term=Ma+LJ&cauthor_id=12712197), [Serge Smirnov](https://pubmed.ncbi.nlm.nih.gov/?term=Smirnov+S&cauthor_id=12712197), [Seth Purcell](https://pubmed.ncbi.nlm.nih.gov/?term=Purcell+S&cauthor_id=12712197), [Bushra Rehman](https://pubmed.ncbi.nlm.nih.gov/?term=Rehman+B&cauthor_id=12712197), [Timothy Elkins](https://pubmed.ncbi.nlm.nih.gov/?term=Elkins+T&cauthor_id=12712197), [Reinhard Engels](https://pubmed.ncbi.nlm.nih.gov/?term=Engels+R&cauthor_id=12712197), [Shunguang Wang](https://pubmed.ncbi.nlm.nih.gov/?term=Wang+S&cauthor_id=12712197), [Cydney B Nielsen](https://pubmed.ncbi.nlm.nih.gov/?term=Nielsen+CB&cauthor_id=12712197), [Jonathan Butler](https://pubmed.ncbi.nlm.nih.gov/?term=Butler+J&cauthor_id=12712197), [Matthew Endrizzi](https://pubmed.ncbi.nlm.nih.gov/?term=Endrizzi+M&cauthor_id=12712197), [Dayong Qui](https://pubmed.ncbi.nlm.nih.gov/?term=Qui+D&cauthor_id=12712197), [Peter Ianakiev](https://pubmed.ncbi.nlm.nih.gov/?term=Ianakiev+P&cauthor_id=12712197), [Deborah Bell-Pedersen](https://pubmed.ncbi.nlm.nih.gov/?term=Bell-Pedersen+D&cauthor_id=12712197), [Mary Anne Nelson](https://pubmed.ncbi.nlm.nih.gov/?term=Nelson+MA&cauthor_id=12712197), [Margaret Werner-Washburne](https://pubmed.ncbi.nlm.nih.gov/?term=Werner-Washburne+M&cauthor_id=12712197), [Claude P Selitrennikoff](https://pubmed.ncbi.nlm.nih.gov/?term=Selitrennikoff+CP&cauthor_id=12712197), [John A Kinsey](https://pubmed.ncbi.nlm.nih.gov/?term=Kinsey+JA&cauthor_id=12712197), [Edward L Braun](https://pubmed.ncbi.nlm.nih.gov/?term=Braun+EL&cauthor_id=12712197), [Alex Zelter](https://pubmed.ncbi.nlm.nih.gov/?term=Zelter+A&cauthor_id=12712197), [Ulrich Schulte](https://pubmed.ncbi.nlm.nih.gov/?term=Schulte+U&cauthor_id=12712197), [Gregory O Kothe](https://pubmed.ncbi.nlm.nih.gov/?term=Kothe+GO&cauthor_id=12712197), [Gregory Jedd](https://pubmed.ncbi.nlm.nih.gov/?term=Jedd+G&cauthor_id=12712197), [Werner Mewes](https://pubmed.ncbi.nlm.nih.gov/?term=Mewes+W&cauthor_id=12712197), [Chuck Staben](https://pubmed.ncbi.nlm.nih.gov/?term=Staben+C&cauthor_id=12712197), [Edward Marcotte](https://pubmed.ncbi.nlm.nih.gov/?term=Marcotte+E&cauthor_id=12712197), [David Greenberg](https://pubmed.ncbi.nlm.nih.gov/?term=Greenberg+D&cauthor_id=12712197), [Alice Roy](https://pubmed.ncbi.nlm.nih.gov/?term=Roy+A&cauthor_id=12712197), [Karen Foley](https://pubmed.ncbi.nlm.nih.gov/?term=Foley+K&cauthor_id=12712197), [Jerome Naylor](https://pubmed.ncbi.nlm.nih.gov/?term=Naylor+J&cauthor_id=12712197), [Nicole Stange-Thomann](https://pubmed.ncbi.nlm.nih.gov/?term=Stange-Thomann+N&cauthor_id=12712197), [Robert Barrett](https://pubmed.ncbi.nlm.nih.gov/?term=Barrett+R&cauthor_id=12712197), [Sante Gnerre](https://pubmed.ncbi.nlm.nih.gov/?term=Gnerre+S&cauthor_id=12712197), [Michael Kamal](https://pubmed.ncbi.nlm.nih.gov/?term=Kamal+M&cauthor_id=12712197), [Manolis Kamvysselis](https://pubmed.ncbi.nlm.nih.gov/?term=Kamvysselis+M&cauthor_id=12712197), [Evan Mauceli](https://pubmed.ncbi.nlm.nih.gov/?term=Mauceli+E&cauthor_id=12712197), [Cord Bielke](https://pubmed.ncbi.nlm.nih.gov/?term=Bielke+C&cauthor_id=12712197), [Stephen Rudd](https://pubmed.ncbi.nlm.nih.gov/?term=Rudd+S&cauthor_id=12712197), [Dmitrij Frishman](https://pubmed.ncbi.nlm.nih.gov/?term=Frishman+D&cauthor_id=12712197), [Svetlana Krystofova](https://pubmed.ncbi.nlm.nih.gov/?term=Krystofova+S&cauthor_id=12712197), [Carolyn Rasmussen](https://pubmed.ncbi.nlm.nih.gov/?term=Rasmussen+C&cauthor_id=12712197), [Robert L Metzenberg](https://pubmed.ncbi.nlm.nih.gov/?term=Metzenberg+RL&cauthor_id=12712197), [David D Perkins](https://pubmed.ncbi.nlm.nih.gov/?term=Perkins+DD&cauthor_id=12712197), [Scott Kroken](https://pubmed.ncbi.nlm.nih.gov/?term=Kroken+S&cauthor_id=12712197), [Carlo Cogoni](https://pubmed.ncbi.nlm.nih.gov/?term=Cogoni+C&cauthor_id=12712197), [Giuseppe Macino](https://pubmed.ncbi.nlm.nih.gov/?term=Macino+G&cauthor_id=12712197), [David Catcheside](https://pubmed.ncbi.nlm.nih.gov/?term=Catcheside+D&cauthor_id=12712197), [Weixi Li](https://pubmed.ncbi.nlm.nih.gov/?term=Li+W&cauthor_id=12712197), [Robert J Pratt](https://pubmed.ncbi.nlm.nih.gov/?term=Pratt+RJ&cauthor_id=12712197), [Stephen A Osmani](https://pubmed.ncbi.nlm.nih.gov/?term=Osmani+SA&cauthor_id=12712197), [Colin P C DeSouza](https://pubmed.ncbi.nlm.nih.gov/?term=DeSouza+CP&cauthor_id=12712197), [Louise Glass](https://pubmed.ncbi.nlm.nih.gov/?term=Glass+L&cauthor_id=12712197), [Marc J Orbach](https://pubmed.ncbi.nlm.nih.gov/?term=Orbach+MJ&cauthor_id=12712197), [J Andrew Berglund](https://pubmed.ncbi.nlm.nih.gov/?term=Berglund+JA&cauthor_id=12712197), [Rodger Voelker](https://pubmed.ncbi.nlm.nih.gov/?term=Voelker+R&cauthor_id=12712197), [Oded Yarden](https://pubmed.ncbi.nlm.nih.gov/?term=Yarden+O&cauthor_id=12712197), [Michael Plamann](https://pubmed.ncbi.nlm.nih.gov/?term=Plamann+M&cauthor_id=12712197), [Stephan Seiler](https://pubmed.ncbi.nlm.nih.gov/?term=Seiler+S&cauthor_id=12712197), [Jay Dunlap](https://pubmed.ncbi.nlm.nih.gov/?term=Dunlap+J&cauthor_id=12712197), [Alan Radford](https://pubmed.ncbi.nlm.nih.gov/?term=Radford+A&cauthor_id=12712197), [Rodolfo Aramayo](https://pubmed.ncbi.nlm.nih.gov/?term=Aramayo+R&cauthor_id=12712197), [Donald O Natvig](https://pubmed.ncbi.nlm.nih.gov/?term=Natvig+DO&cauthor_id=12712197), [Lisa A Alex](https://pubmed.ncbi.nlm.nih.gov/?term=Alex+LA&cauthor_id=12712197), [Gertrud Mannhaupt](https://pubmed.ncbi.nlm.nih.gov/?term=Mannhaupt+G&cauthor_id=12712197), [Daniel J Ebbole](https://pubmed.ncbi.nlm.nih.gov/?term=Ebbole+DJ&cauthor_id=12712197), [Michael Freitag](https://pubmed.ncbi.nlm.nih.gov/?term=Freitag+M&cauthor_id=12712197), [Ian Paulsen](https://pubmed.ncbi.nlm.nih.gov/?term=Paulsen+I&cauthor_id=12712197), [Matthew S Sachs](https://pubmed.ncbi.nlm.nih.gov/?term=Sachs+MS&cauthor_id=12712197), [Eric S Lander](https://pubmed.ncbi.nlm.nih.gov/?term=Lander+ES&cauthor_id=12712197), [Chad Nusbaum](https://pubmed.ncbi.nlm.nih.gov/?term=Nusbaum+C&cauthor_id=12712197), [Bruce Birren](https://pubmed.ncbi.nlm.nih.gov/?term=Birren+B&cauthor_id=12712197).2003.The genome sequence of the filamentous fungus Neurospora crassa.Nature. 2003 Apr 24;422:859-68.

[Jasper R L Depotter](https://pubmed.ncbi.nlm.nih.gov/?term=Depotter+JRL&cauthor_id=31282048),[Xiaoqian Shi-Kunne](https://pubmed.ncbi.nlm.nih.gov/?term=Shi-Kunne+X&cauthor_id=31282048),[Hélène Missonnier](https://pubmed.ncbi.nlm.nih.gov/?term=Missonnier+H&cauthor_id=31282048) ,[Tingli Liu](https://pubmed.ncbi.nlm.nih.gov/?term=Liu+T&cauthor_id=31282048),[Luigi Faino](https://pubmed.ncbi.nlm.nih.gov/?term=Faino+L&cauthor_id=31282048),[Grardy C M van den Berg](https://pubmed.ncbi.nlm.nih.gov/?term=van+den+Berg+GCM&cauthor_id=31282048),[Thomas A Wood](https://pubmed.ncbi.nlm.nih.gov/?term=Wood+TA&cauthor_id=31282048),[Baolong Zhang](https://pubmed.ncbi.nlm.nih.gov/?term=Zhang+B&cauthor_id=31282048),[Alban Jacques](https://pubmed.ncbi.nlm.nih.gov/?term=Jacques+A&cauthor_id=31282048),[Michael F Seidl](https://pubmed.ncbi.nlm.nih.gov/?term=Seidl+MF&cauthor_id=31282048),[Bart P H J Thomma](https://pubmed.ncbi.nlm.nih.gov/?term=Thomma+BPHJ&cauthor_id=31282048). 2019. Dynamic virulence-related regions of the plant pathogenic fungus Verticillium dahliae display enhanced sequence conservation.Molecular ecology 28:3482-3495.

[Jeremy Semeiks](https://pubmed.ncbi.nlm.nih.gov/?term=Semeiks+J&cauthor_id=25015739), [Dominika Borek](https://pubmed.ncbi.nlm.nih.gov/?term=Borek+D&cauthor_id=25015739),[Zbyszek Otwinowski](https://pubmed.ncbi.nlm.nih.gov/?term=Otwinowski+Z&cauthor_id=25015739),[Nick V Grishin](https://pubmed.ncbi.nlm.nih.gov/?term=Grishin+NV&cauthor_id=25015739). 2014.Comparative genome sequencing reveals chemotype-specific gene clusters in the toxigenic black mold Stachybotrys.BMC Genomics 15:590.

[Mario E E Franco](https://pubmed.ncbi.nlm.nih.gov/?term=Franco+MEE&cauthor_id=34797921), [Jennifer H Wisecaver](https://pubmed.ncbi.nlm.nih.gov/?term=Wisecaver+JH&cauthor_id=34797921), [A Elizabeth Arnold](https://pubmed.ncbi.nlm.nih.gov/?term=Arnold+AE&cauthor_id=34797921), [Yu-Ming Ju](https://pubmed.ncbi.nlm.nih.gov/?term=Ju+YM&cauthor_id=34797921) , [Jason C Slot](https://pubmed.ncbi.nlm.nih.gov/?term=Slot+JC&cauthor_id=34797921), [Steven Ahrendt](https://pubmed.ncbi.nlm.nih.gov/?term=Ahrendt+S&cauthor_id=34797921) , [Lillian P Moore](https://pubmed.ncbi.nlm.nih.gov/?term=Moore+LP&cauthor_id=34797921),[Katharine E Eastman](https://pubmed.ncbi.nlm.nih.gov/?term=Eastman+KE&cauthor_id=34797921), [Kelsey Scott](https://pubmed.ncbi.nlm.nih.gov/?term=Scott+K&cauthor_id=34797921), [Zachary Konkel](https://pubmed.ncbi.nlm.nih.gov/?term=Konkel+Z&cauthor_id=34797921),[Stephen J Mondo](https://pubmed.ncbi.nlm.nih.gov/?term=Mondo+SJ&cauthor_id=34797921),[Alan Kuo](https://pubmed.ncbi.nlm.nih.gov/?term=Kuo+A&cauthor_id=34797921),[Richard D Hayes](https://pubmed.ncbi.nlm.nih.gov/?term=Hayes+RD&cauthor_id=34797921),[Sajeet Haridas](https://pubmed.ncbi.nlm.nih.gov/?term=Haridas+S&cauthor_id=34797921),[Bill Andreopoulos](https://pubmed.ncbi.nlm.nih.gov/?term=Andreopoulos+B&cauthor_id=34797921),[Robert Riley](https://pubmed.ncbi.nlm.nih.gov/?term=Riley+R&cauthor_id=34797921),[Kurt LaButti](https://pubmed.ncbi.nlm.nih.gov/?term=LaButti+K&cauthor_id=34797921),[Jasmyn Pangilinan](https://pubmed.ncbi.nlm.nih.gov/?term=Pangilinan+J&cauthor_id=34797921),[Anna Lipzen](https://pubmed.ncbi.nlm.nih.gov/?term=Lipzen+A&cauthor_id=34797921), [Mojgan Amirebrahimi](https://pubmed.ncbi.nlm.nih.gov/?term=Amirebrahimi+M&cauthor_id=34797921),[Juying Yan](https://pubmed.ncbi.nlm.nih.gov/?term=Yan+J&cauthor_id=34797921),[Catherine Adam](https://pubmed.ncbi.nlm.nih.gov/?term=Adam+C&cauthor_id=34797921), [Keykhosrow Keymanesh](https://pubmed.ncbi.nlm.nih.gov/?term=Keymanesh+K&cauthor_id=34797921),[Vivian Ng](https://pubmed.ncbi.nlm.nih.gov/?term=Ng+V&cauthor_id=34797921)., [Katherine Louie](https://pubmed.ncbi.nlm.nih.gov/?term=Louie+K&cauthor_id=34797921),[Trent Northen](https://pubmed.ncbi.nlm.nih.gov/?term=Northen+T&cauthor_id=34797921),[Elodie Drula](https://pubmed.ncbi.nlm.nih.gov/?term=Drula+E&cauthor_id=34797921),[Bernard Henrissat](https://pubmed.ncbi.nlm.nih.gov/?term=Henrissat+B&cauthor_id=34797921),[Huei-Mei Hsieh](https://pubmed.ncbi.nlm.nih.gov/?term=Hsieh+HM&cauthor_id=34797921),[Ken Youens-Clark](https://pubmed.ncbi.nlm.nih.gov/?term=Youens-Clark+K&cauthor_id=34797921),[François Lutzoni](https://pubmed.ncbi.nlm.nih.gov/?term=Lutzoni+F&cauthor_id=34797921),[Jolanta Miadlikowska](https://pubmed.ncbi.nlm.nih.gov/?term=Miadlikowska+J&cauthor_id=34797921),[Daniel C Eastwood](https://pubmed.ncbi.nlm.nih.gov/?term=Eastwood+DC&cauthor_id=34797921),[Richard C Hamelin](https://pubmed.ncbi.nlm.nih.gov/?term=Hamelin+RC&cauthor_id=34797921), [Igor V Grigoriev](https://pubmed.ncbi.nlm.nih.gov/?term=Grigoriev+IV&cauthor_id=34797921),[Jana M U'Ren](https://pubmed.ncbi.nlm.nih.gov/?term=U%27Ren+JM&cauthor_id=34797921). 2022. Ecological generalism drives hyperdiversity of secondary metabolite gene clusters in xylarialean endophytes.New Phytol 233:1317-1330.

[Matt T Kasson](https://pubmed.ncbi.nlm.nih.gov/?term=Kasson+MT&cauthor_id=30701257), [Lindsay R Kasson](https://pubmed.ncbi.nlm.nih.gov/?term=Kasson+LR&cauthor_id=30701257) , [Kristen L Wickert](https://pubmed.ncbi.nlm.nih.gov/?term=Wickert+KL&cauthor_id=30701257), [Donald D Davis](https://pubmed.ncbi.nlm.nih.gov/?term=Davis+DD&cauthor_id=30701257) , [Jason E Stajich](https://pubmed.ncbi.nlm.nih.gov/?term=Stajich+JE&cauthor_id=30701257). 2019. Genome Sequence of a Lethal Vascular Wilt Fungus, Verticillium nonalfalfae, a Biological Control Used Against the Invasive Ailanthus altissima. Microbiol Resour Announc 8:e01619-18.

[Peng Zheng](https://pubmed.ncbi.nlm.nih.gov/?term=Zheng+P&cauthor_id=22112802), [YongliangXia](https://pubmed.ncbi.nlm.nih.gov/?term=Xia+Y&cauthor_id=22112802), [GuohuaXiao](https://pubmed.ncbi.nlm.nih.gov/?term=Xiao+G&cauthor_id=22112802), [ChenghuiXiong](https://pubmed.ncbi.nlm.nih.gov/?term=Xiong+C&cauthor_id=22112802), [XiaoHu](https://pubmed.ncbi.nlm.nih.gov/?term=Hu+X&cauthor_id=22112802),[SiweiZhang](https://pubmed.ncbi.nlm.nih.gov/?term=Zhang+S&cauthor_id=22112802), [Huajun Zheng](https://pubmed.ncbi.nlm.nih.gov/?term=Zheng+H&cauthor_id=22112802), [YinHuang](https://pubmed.ncbi.nlm.nih.gov/?term=Huang+Y&cauthor_id=22112802), [YanZhou](https://pubmed.ncbi.nlm.nih.gov/?term=Zhou+Y&cauthor_id=22112802), [Shengyue Wang](https://pubmed.ncbi.nlm.nih.gov/?term=Wang+S&cauthor_id=22112802), [Guo-Ping Zhao](https://pubmed.ncbi.nlm.nih.gov/?term=Zhao+GP&cauthor_id=22112802), [Xingzhong Liu](https://pubmed.ncbi.nlm.nih.gov/?term=Liu+X&cauthor_id=22112802), [Raymond J, StLeger](https://pubmed.ncbi.nlm.nih.gov/?term=St+Leger+RJ&cauthor_id=22112802), [Chengshu Wang](https://pubmed.ncbi.nlm.nih.gov/?term=Wang+C&cauthor_id=22112802).2011.Genome sequence of the insect pathogenic fungus Cordyceps militaris, a valued traditional Chinese medicine.Genome Biol. 2011 Nov 23;12,R116.

[Qiang,Gao](https://pubmed.ncbi.nlm.nih.gov/?term=Gao+Q&cauthor_id=21253567), [Kai Jin](https://pubmed.ncbi.nlm.nih.gov/?term=Jin+K&cauthor_id=21253567), [Sheng-Hua Ying](https://pubmed.ncbi.nlm.nih.gov/?term=Ying+SH&cauthor_id=21253567), [Yongjun Zhang](https://pubmed.ncbi.nlm.nih.gov/?term=Zhang+Y&cauthor_id=21253567), [GuohuaXiao](https://pubmed.ncbi.nlm.nih.gov/?term=Xiao+G&cauthor_id=21253567), [YanfangShang](https://pubmed.ncbi.nlm.nih.gov/?term=Shang+Y&cauthor_id=21253567), [Zhibing Duan](https://pubmed.ncbi.nlm.nih.gov/?term=Duan+Z&cauthor_id=21253567), [XiaoHu](https://pubmed.ncbi.nlm.nih.gov/?term=Hu+X&cauthor_id=21253567), [Xue-QinXie](https://pubmed.ncbi.nlm.nih.gov/?term=Xie+XQ&cauthor_id=21253567), [Gang Zhou](https://pubmed.ncbi.nlm.nih.gov/?term=Zhou+G&cauthor_id=21253567), [Guoxiong Peng](https://pubmed.ncbi.nlm.nih.gov/?term=Peng+G&cauthor_id=21253567), [ZhibingLuo](https://pubmed.ncbi.nlm.nih.gov/?term=Luo+Z&cauthor_id=21253567), [Wei Huang](https://pubmed.ncbi.nlm.nih.gov/?term=Huang+W&cauthor_id=21253567), [Bing Wang](https://pubmed.ncbi.nlm.nih.gov/?term=Wang+B&cauthor_id=21253567), [WeiguoFang](https://pubmed.ncbi.nlm.nih.gov/?term=Fang+W&cauthor_id=21253567), [SibaoWang](https://pubmed.ncbi.nlm.nih.gov/?term=Wang+S&cauthor_id=21253567), [Yi Zhong](https://pubmed.ncbi.nlm.nih.gov/?term=Zhong+Y&cauthor_id=21253567), [Li-Jun Ma](https://pubmed.ncbi.nlm.nih.gov/?term=Ma+LJ&cauthor_id=21253567), [Raymond J, St Leger](https://pubmed.ncbi.nlm.nih.gov/?term=St+Leger+RJ&cauthor_id=21253567), [Guo-PingZhao](https://pubmed.ncbi.nlm.nih.gov/?term=Zhao+GP&cauthor_id=21253567), [Yan Pei](https://pubmed.ncbi.nlm.nih.gov/?term=Pei+Y&cauthor_id=21253567), [Ming-Guang Feng](https://pubmed.ncbi.nlm.nih.gov/?term=Feng+MG&cauthor_id=21253567), [Yuxian Xia](https://pubmed.ncbi.nlm.nih.gov/?term=Xia+Y&cauthor_id=21253567), [Chengshu Wang](https://pubmed.ncbi.nlm.nih.gov/?term=Wang+C&cauthor_id=21253567).2011.PLoS Genet. 2011 Jan 6;7,e1001264.

Schardl, C.L., Young, C.A., Hesse, U., Amyotte, S.G., Andreeva, K., Calie, P.J., Fleetwood, D.J., Haws, D.C., Moore, N., Oeser, B., 2013. Plant-symbiotic fungi as chemical engineers: multi-genome analysis of the Clavicipitaceae reveals dynamics of alkaloid loci. PLoS genetics 9, e1003323.

Sun, Z.B., Sun, M.H., Li, S.D., 2015. Draft Genome Sequence of Mycoparasite *Clonostachys rosea* Strain 67-1. Genome Announcement 3(3), e00546-15.

Terfehr, D., Dahlmann, T.A., Specht, T., Zadra, I., Kürnsteiner, H., Kück, U., 2014. Genome sequence and annotation of Acremonium chrysogenum, producer of the β-lactam antibiotic cephalosporin C. Genome announcements 2, e00948-00914.

Xiao, G., Ying, S.-H., Zheng, P., Wang, Z.-L., Zhang, S., Xie, X.-Q., Shang, Y., St Leger, R.J., Zhao, G.-P., Wang, C., 2012. Genomic perspectives on the evolution of fungal entomopathogenicity in Beauveria bassiana. Scientific reports 2, 1-10.

[Yamini Agrawal](https://pubmed.ncbi.nlm.nih.gov/?term=Agrawal+Y&cauthor_id=27189621) , [TarunNarwani](https://pubmed.ncbi.nlm.nih.gov/?term=Narwani+T&cauthor_id=27189621), [Srikrishna Subramanian](https://pubmed.ncbi.nlm.nih.gov/?term=Subramanian+S&cauthor_id=27189621) .2016.Genome sequence and comparative analysis of clavicipitaceous insect-pathogenic fungus Aschersonia badia with Metarhizium spp.BMC Genomics. 2016 May 17;17:367.

[Yi Li](https://pubmed.ncbi.nlm.nih.gov/?term=Li+Y&cauthor_id=27343682) , [Tom Hsiang](https://pubmed.ncbi.nlm.nih.gov/?term=Hsiang+T&cauthor_id=27343682) , [Rui-Heng Yang](https://pubmed.ncbi.nlm.nih.gov/?term=Yang+RH&cauthor_id=27343682) , [Xiao-Di Hu](https://pubmed.ncbi.nlm.nih.gov/?term=Hu+XD&cauthor_id=27343682) ,[Ke Wang](https://pubmed.ncbi.nlm.nih.gov/?term=Wang+K&cauthor_id=27343682) ,[Wen-Jing Wang](https://pubmed.ncbi.nlm.nih.gov/?term=Wang+WJ&cauthor_id=27343682) ,[Xiao-Liang Wang](https://pubmed.ncbi.nlm.nih.gov/?term=Wang+XL&cauthor_id=27343682),[Lei Jiao](https://pubmed.ncbi.nlm.nih.gov/?term=Jiao+L&cauthor_id=27343682),[Yi-Jian Yao](https://pubmed.ncbi.nlm.nih.gov/?term=Yao+YJ&cauthor_id=27343682).2016. Comparison of different sequencing and assembly strategies for a repeat-rich fungal genome, Ophiocordyceps sinensis. J Microbiol Methods 128:1-6.
