# Supplementary material for: The genome and transcriptome of Sarocladium terricola provide insight into ergosterol biosynthesis
Source: Front Cell Infect Microbiol. 2023 Apr 14;13:1181287. doi: 10.3389/fcimb.2023.1181287 (PMC10140317; doi:10.3389/fcimb.2023.1181287)
Supplement: Supplementary file 2 [file Table_2.docx]

Table S2. Transcriptomes information of *Sarocladium terricola* B106.

| Group | Sample | Total clean reads | Total clean bases (Gb) | Clean reads Q30 (%) | Clean reads ratio (%) | Total mapping ratio (%) | Total gene number |
| --- | --- | --- | --- | --- | --- | --- | --- |
| HC-1h | 106Huang1 | 40,086,050 | 6.01 | 96.30 | 97.98 | 98.18 | 9,176 |
| HC-1h | 106Huang2 | 37,043,298 | 5.56 | 95.93 | 98.16 | 98.59 | 9,738 |
| HC-1h | 106Huang3 | 42,033,580 | 6.31 | 96.45 | 98.28 | 98.33 | 9,219 |
| HC-1t | 106Tu1 | 39,804,426 | 5.97 | 96.41 | 98.35 | 97.65 | 9,374 |
| HC-1t | 106Tu2 | 38,454,078 | 5.77 | 96.42 | 98.19 | 97.96 | 9,402 |
| HC-1t | 106Tu3 | 38,781,554 | 5.82 | 96.45 | 98.21 | 98.16 | 9,457 |
