# Supplementary figures and images for: The genome and transcriptome of Sarocladium terricola provide insight into ergosterol biosynthesis
Source: Front Cell Infect Microbiol. 2023 Apr 14;13:1181287. doi: 10.3389/fcimb.2023.1181287 (PMC10140317; doi:10.3389/fcimb.2023.1181287)

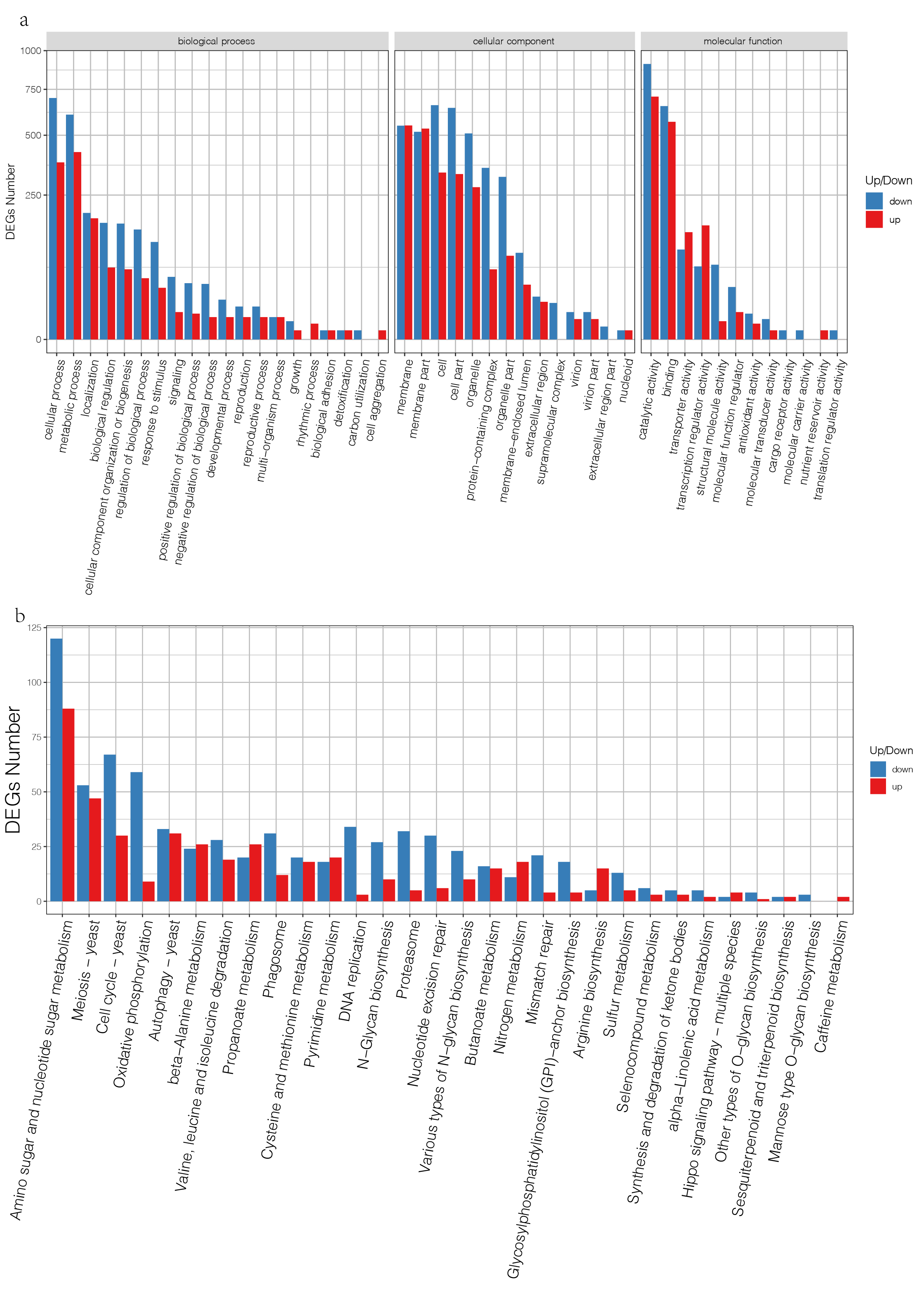

Supplement: Supplementary Figure 1 — GO function and KEEG pathway enrichment analyses in responding to the two type media of S. terricola. (A) differentially expressed genes number of the most enriched GO term, (B) differentially expressed genes number of the most enriched KEGG pathway. [file Image_1.jpeg]
